# Nonmedical Use of Benzodiazepines among Immigrant and Native-Born Adolescents in Spain: National Trends and Related Factors

**DOI:** 10.3390/ijerph18031171

**Published:** 2021-01-28

**Authors:** Pilar Carrasco-Garrido, Dania Rocío Díaz Rodríguez, Isabel Jiménez-Trujillo, Valentín Hernández-Barrera, Lidiane Lima Florencio, Domingo Palacios-Ceña

**Affiliations:** 1 Preventive Medicine and Public Health Area, Universidad Rey Juan Carlos Av., Atenas s/n, Alcorcón, 28922 Madrid, Spain; isabel.jimenez@urjc.es (I.J.-T.); valentin.hernandez@urjc.es (V.H.-B.); 2 Hospital Universitario del Henares, Av. de Marie Curie s/n, Coslada, 28822 Madrid, Spain; drocio.diaz@salud.madrid.org; 3 Department of Physical Therapy, Occupational Therapy, Rehabilitation and Physical Medicine, Universidad Rey Juan Carlos Av., Atenas s/n, Alcorcón, 28922 Madrid, Spain; lidiane.florencio@urjc.es (L.L.F.); domingo.palacios@urjc.es (D.P.-C.)

**Keywords:** nonmedical use, benzodiazepines, immigrant, student population, adolescents, drug survey

## Abstract

Background: the nonmedical use of prescribed medications among adolescents has increased significantly in recent years. We aimed to identify the patterns of benzodiazepine nonmedical use and its evolution during the decade 2006–2016 among immigrant and native-born adolescent populations. Methods: we used individualized secondary data retrieved from the 2006–2016 Spanish State Survey on Drug Use in Secondary Education (ESTUDES) of the school-aged population. Using logistic multivariate regression models, we estimated the independent effect of each of these variables on nonmedical use. Two models were generated: one for immigrant adolescents and one for native-born adolescents. Results: during the decade 2006–2016, 2.81% of native-born and 3.36% of immigrant adolescent students made nonmedical use of benzodiazepines. Gender and socioeconomic status were found to be related to the nonmedical use of benzodiazepines. Consumption of illegal psychoactive substances, other than marijuana, was the variable of greatest value (aOR = 6.00, 95% CI 3.89–9.27). Perceived risks and drug availability were found to be predictors for the nonmedical use of benzodiazepines in both immigrant and native-born adolescents. Conclusion: in Spain, patterns of benzodiazepine nonmedical use among immigrant and native-born adolescents are similar. The results of this study refute certain stereotypes related to consumption of substances among immigrant adolescents, identifying them as a risk group.

## 1. Introduction

Adolescence is a time for learning, skill acquisition, and experimentation and, as such, sets the stage for a large portion of behavior later in adult life; but it is also a time at high risk for the onset of psychoactive substance consumption.

Nonmedical use of prescription drugs has increased significantly among our younger population [1]. Recently, a report from the U.S. entitled “Monitoring the Future” indicated that the abuse of prescription drugs for nonmedical use reached values of 4.7% among adolescents aged 12 to 17 [2], with psychedelic drugs the most frequently used. The last European School Survey Project on Alcohol and Other Drugs (ESPAD) showed a 6% rate of nonmedical use of tranquilizers and sedatives among European adolescents [3]. Furthermore, a recent study on U.S. high school seniors found that 7.5% of these students had made nonmedical use of anxiolytics at least once in their lifetimes [4].

Currently, European adolescents from migrant families represent one-quarter of all school-aged adolescents [5]. The associations between the different factors influencing psychoactive substance consumption patterns among adolescents with migratory backgrounds have been studied. However, most of these studies have assessed the role of linguistic, psychological and acculturation factors with regard to the phenomenon of cultural change and adaptation that takes place when individuals from different cultures come into contact with each other [6]. Even so, research comparing the use of legal and illegal psychoactive substances among native-born and immigrant adolescents is scarce. Some studies performed in Spain, Germany, and the United States [7,8,9] have found differences in psychoactive substance consumption patterns among immigrant and native-born adolescents and in the acculturation process among immigrant adolescents.

It should be noted that study results are not homogeneous. Although certain studies point to greater consumption among immigrant adolescents, other studies, such as one carried out with school populations in six European Union countries [10], showed that the consumption of certain psychoactive substances was higher among native-born adolescents.

Even though the influence of demographic and social characteristics on the nonmedical use of prescription drugs among the younger population has been described, there are not many studies comparing exactly how psychoactive drug use occurs among immigrant and native-born adolescents [11,12].

In recent years, immigrant adolescents in Spain have become new protagonists of the Spanish migratory reality. This population is receiving increasing attention in all the areas where they have been incorporated: education, health, work, and the consumption of substances. However, in our country there are few studies that have compared the recreational use of prescription drugs.

It is in this context that we carried out a study to identify the patterns of benzodiazepine nonmedical use and to estimate trends over time regarding prevalence during the decade 2006–2016 among the immigrant and native-born adolescent populations.

## 2. Materials and Methods

### 2.1. Data Source

We conducted a nationwide, longitudinal study with six repeated cross-sectional studies over time—also known as a pooled cross-sectional times series model—based on individualized secondary data gathered in the Spanish National Drug Plan’s Spanish State Survey on Drug Use in Secondary Education (ESTUDES) from the school-aged population of both sexes between 2006 and 2016 [13]. The Spanish National Drug Plan carries out a biannual national survey (ESTUDES) of students aged 14 to 18 who are in the third and fourth years of the mandatory state study program, first and second years of pre-university courses, and mid-grade training cycles of the Spanish National Vocational Qualification in both public and private centers. The survey is performed in order to determine current use and trends in drug consumption among students aged 14 to 18 years using a standardized, anonymous, and self-administered written questionnaire.

Sampling was two-stage and by conglomerates. Schools were selected at random as first-stage units while classrooms became second-stage units. All students in the selected classrooms were subsequently included in order to simplify the sample design, application, and analysis. The study population consisted of a total of 188,962 students of both sexes, aged 14 to 18 years, and residing in Spain during the decade 2006–2016 (26,454 from ESTUDES 2006; 30,183 from ESTUDES 2008; 31,967 from ESTUDES 2010; 27,503 from ESTUDES 2012; 37,486 from ESTUDES 2014; and 35,639 from ESTUDES 2016).

Native or immigrant status was based on the country where the adolescents were born.

The dependent variable for this study was the dichotomous answer “yes” or “no” to the question “Have you taken a tranquilizer, sedative, and/or sleeping pill without a prescription during the last 30 days?” Independent variables collected in the study were the primary sociodemographic characteristics of the students and their parents (mother’s and father’s occupation and parents’ education) and variables related to the use of other legal drugs (alcohol and tobacco in the previous 30 days) and illegal psychoactive substances (marijuana and drugs other than marijuana (such as LSD, amphetamines, cocaine, and heroin)) in the previous year.

Other factors we analyzed included risk perception for nonmedical use of drugs, the availability of these substances, and the information adolescents had received. Details of the ESTUDES methodology have been previously published in a related article [14].

### 2.2. Statistical Analysis

We calculated the prevalence of the nonmedical use of benzodiazepines for each of the six surveys according to the study variables. The selection process began with univariate analysis of each variable (age, sex, nationality, occupational status of parents, educational level, alcohol use, tobacco use, marijuana use, illicit psychoactive drug use, perceived health risks, perceived availability and information from school and from each survey.

For the second step, we chose variables for which the univariate test had a *p*-value < 0.25. The last step used the enter method. We selected those variables for which the Wald test had a *p*-value < 0.05. All models were adjusted according to the variables included in the models. The bivariate analysis of the yearly changes was based on a generalized linear model with a binomial response and logit link function (logistic regression) with the year as the continuous variable. All calculations were applied to the native-born and immigrant scholar populations.

In order to estimate the independent effect of each of these variables on the study population (immigrant and native-born adolescents), crude odds ratio (OR) and adjusted odds ratio (aOR) were calculated, together with their corresponding 95% confidence intervals (95% CI), through multivariate analysis techniques and using logistic regression models.

Two models were generated: one for immigrant adolescents and one for native-born adolescents.

Once the models were constructed, we analyzed trends in benzodiazepine nonmedical use during the study period and calculated the crude and adjusted odds ratios. Global trends were estimated using a logistic regression model in which the trend was calculated by introducing the year as a continuous variable and all the other factors as independent variables.

Estimates were made using the svy function (survey command) of the STATA program (STATA Corp, College Station, TX, USA), which allowed us to incorporate the sampling design and weights into all statistical calculations (descriptive, χ2, and logistic regression). Statistical significance was established as a two-tailed α = 0.05.

## 3. Results

The distribution of the various sociodemographic characteristics of both populations is shown in Table 1.

Our results were obtained from 9062 native-born and 1082 immigrant adolescents from different ethnicities (53% Latin American, 18% European, and 13% African).

The data from Table 2 show that during the decade from 2006 to 2016, 2.81% of native-born and 3.36% of immigrant students made nonmedical use of benzodiazepine.

Results of the multivariate analysis performed using logistic regression models (Table 3) show that adolescent girls were more likely to make nonmedical use of these drugs, with values higher among immigrant adolescents. Having unemployed parents significantly increased the probability of making nonmedical use of these drugs in both study populations.

Consuming other legal psychoactive drugs (tobacco, alcohol) was significantly associated with the nonmedical use of benzodiazepines in both study populations.

Regarding illicit drug use, marijuana consumption was significantly related to nonmedical use of benzodiazepines only among native-born individuals (aOR 1.48 95%CI 1.30–1.69). Students who used other types of illicit drugs were more likely to make nonmedical use of these drugs and this association was higher among immigrants (aOR = 6.00, 95% CI 3.89–9.27).

Perceived risks, drug availability, and the amount of information received at school acted as predictors for the nonmedical use of benzodiazepines.

Analysis of trends for nonmedical use of benzodiazepine during 2006–2016 revealed that the OR was 1.21 (95% CI 1.08–1.33). When potential confounders were controlled for, this statistical significance remained unchanged (aOR 1.04; 95% CI 1.03–1.06), which means that there was a significant 4% increase in the nonmedical use of this medication from 2006 to 2016 among native-born students. Among immigrant adolescent students, no significant association was found when analyzing the benzodiazepine nonmedical use trend during the study decade (aOR = 1.04, 95% CI 1.00–1.09)

## 4. Discussion

This article is one of the first to describe and compare the factors related to nonmedical use of benzodiazepines in immigrant and native-born adolescents residing in Spain.

During the study decade, around 3% of the student population made nonmedical use of these drugs (2.81% native-born vs. 3.36% immigrant adolescents). Consumption increased 4% during this decade, although only among native-born students. Our values are higher than the 1.6% found in a recent study performed with data from the American National Survey on Drug Use and Health in an adolescent population [15], but lower than the 7.5% of benzodiazepine nonmedical use found in McCabe’s research on U.S. high school seniors [4]. Furthermore, they are lower than results of the study by Kokkevi et al. undertaken with data from the ESPAD survey for adolescent students from 31 European countries, showing a 5.6% prevalence of nonmedical use of tranquilizers and sedatives [16].

The results show that, in Spain, the patterns of benzodiazepine nonmedical use among immigrant and native-born adolescents are similar. Gender and socioeconomic status had an effect on nonmedical use of these drugs. Adolescent females were twice as likely to make nonmedical use of these drugs as males, coinciding with the results from other studies that indicated that female gender was associated with nonprescription use of tranquilizers/sedatives [17,18]. Females use legal and socially accepted psychoactive drugs such as psychotropics. Due to the double aspect of psychotropic drugs (being substances for both legal and illegal consumption), they are an atypical substance when compared to other drugs. The fact that benzodiazepines are legal psychoactive substances entails the possible consequence that females are more likely to opt for this type of substance.

In our study, adolescents from low socioeconomic status families due to parental unemployment were at greater risk for recreational use of these drugs [19,20]. Adolescents living in poorer socioeconomic conditions face greater problems when making decisions about drug use, as they may have fewer opportunities, less parental supervision, and more stressful events in their lives.

Just as in other studies [14,21], we found that, in Spain, benzodiazepine nonmedical use among immigrant and native-born adolescents was associated with tobacco and alcohol consumption. The present study also found adolescents using tobacco to be at higher risk for nonmedical use of benzodiazepines. This is not surprising because most of these individuals abuse substances and are thus less likely to perceive nonmedical use as being as harmful as the abuse of other substances.

Regarding the use of other psychoactive substances, such as marijuana, our results show that native-born adolescents who made nonmedical use of benzodiazepines were more likely to use marijuana (aOR = 1.48; 95% CI 1.30–1.69) than immigrant adolescents. A recent study performed on a North American school population found that 59% of the adolescents who made nonmedical use of tranquilizers also participated in marijuana co-ingestion [22]; furthermore, this concomitant use often continues into young adulthood, further aggravating the public health problem [23]. Novak et al., in their study based on data from a cross-national investigation of nonmedical prescription drug use in five European countries, found that 28% of those who had misused sedatives in the previous year had also consumed illicit drugs including marijuana [24].

The “immigrant paradox”, whereby immigrants report better health results when compared to native-born persons during their first years in their destination country, did not apply for adolescent immigrants residing in Spain. The nonmedical use of benzodiazepines in immigrant adolescents, which was higher than that of native-born adolescents, may be explained, the disadvantaged socioeconomic position of their parents notwithstanding, by factors derived from the migration process when it is imposed on them by parents, reflecting disaffection because of the time of separation and the changes in social and economic status resulting from the multiple sharp edges of migration [25].

When the low risk perception associated with nonmedical use of benzodiazepines is added together with the ease with which students are able to obtain these drugs, according to statements made by Spanish adolescents, a really disturbing scenario unfolds. Given this situation, it would seem to be essential to identify the factors that favor this low perception of risks and the consequent increase in rates for this addictive behavior among adolescents [26,27]. Adolescents report that benzodiazepines are very easy to acquire. Patients should be informed of the risks of sharing a prescription for tranquilizers and sedatives with other members of the family. Pharmacists can also play an important role in preventing the nonmedical use of these substances by educating people about the exclusivity of prescriptions and the problem of inadequate storage of medicines at home.

Additionally, it should be noted that such similarity in nonmedical use patterns among immigrant and native-born adolescents could be associated with the acculturation of immigrant adolescents, who assimilate the norms, values, and cultural practices of the host country and tend to adopt consumption patterns similar to those that predominate in their new country [28].

In Spain, there are few studies describing benzodiazepine nonmedical use patterns among immigrant and native-born adolescents with a representative sample at the national level; this is the strength of this investigation.

However, our study is limited by the nature of the ESTUDES surveys. The biases of this study are the ETUDES surveys’ cross-sectional character and self-declared data, which could undervalue the answers provided by the study adolescents. This is because the information obtained during interviews was subjective and may be subject to recall errors or the tendency of subjects to give socially desirable responses in interviews.

## 5. Conclusions

In Spain, 2.81% of native-born students and 3.36% of immigrant students made nonmedical use of benzodiazepines. Consumption increased 4% in just one decade, although only among the native-born. Patterns for nonmedical use of benzodiazepines among immigrant and native-born adolescents in our country were similar. Gender, socioeconomic status, legal and illegal psychoactive drug use, perceived risks, and the availability of these drugs were the factors defining the nonmedical use profile of adolescents in Spain.

The results of this study refute certain stereotypes related to consumption of substances among immigrant adolescents and identify them as a risk group. However, the findings present evidence that the nonmedical use of benzodiazepines in Spain is a problematic form of behavior among adolescents, regardless of whether they are immigrant or native-born. Our study provides interesting and valuable results that could be applied toward prevention measures against nonmedical use of psychoactive substances, such as benzodiazepines, among the immigrant and native-born school populations. We believe that these results can effectively contribute to the development of prevention programs for drug use in adolescence, as mandated by its significant increase in just one decade.

## Figures and Tables

**Table 1:** Sociodemographic variables of the study population. ESTUDES surveys 2006–2016.

		Native-Born	Immigrants
		N (%)	N (%)
Sex	Male	83,971 (49.65)	9285 (47.44)
	Female	85,141 (50.35)	10,287 (52.56)
Age	14–15	74,197 (43.87)	7439 (38.01)
	16–18	94,915 (56.13)	12,134 (61.99)
Occupational Status of Parents	Unemployed	5921 (3.5)	1467 (7.5)
	Employed	32,280 (19.09)	6119 (31.26)
	Inactive	130,912 (77.41)	11,986 (61.24)
Educational Level of Parents	No formal education	4798 (3.36)	1151 (7.37)
	Primary school	52,980 (37.07)	4904 (31.41)
	Secondary school	34,268 (23.97)	4096 (26.23)
	Higher education	50,889 (35.6)	5463 (34.99)
Total		169,112 (100)	19,572 (100)

**Table 2:** Prevalence of nonmedical use of benzodiazepines in native-born and immigrant adolescents in Spain. ESTUDES Surveys.

	2006	2008	2010	2012	2014	2016	Combined Years	^a^ Linear Trend *p*-Value
	n (%) CI 95%	n (%) CI 95%	n (%) CI 95%	n (%) CI 95%	n (%) CI 95%	n (%) CI 95%	n (%) CI 95%	
**Native-Born**								
Consumption *	890 (3.63) (3.32–3.98)	1.358 (5.03) (4.66–5.44)	1.397 (4.9) (4.58–5.24)	1.574 (6.46) (6.08–6.86)	2.006 (6.04) (5.74–6.35)	1.836 (5.82) (5.55–6.16)	9.062 (5.36) (5.22–5.50)	0.000
Nonmedical Use	600 (2.45) (2.19–2.74)	753 (2.79) (2.50–3.10)	824 (2.89) (2.64–3.16)	806 (3.31) (3.03–3.61)	953 (2.87) (2.66–3.09)	820 (2.6) (2.39–2.83)	4.756 (2.81) (2.71–2.92)	0.342
**Immigrant**								
Consumption *	63 (3.25) (2.30–4.56)	172 (5.48) (4.53–6.62)	164 (4.81) (4.07–5.67)	219 (6.99) (5.96–8.19)	274 (6.46) (5.60–7.45)	190 (5.12) (4.31–6.07)	1.082 (5.53) (5.14–5.94)	0.007
Nonmedical Use	44 (2.26) (1.52–3.34)	109 (3.48) (2.74–4.42)	127 (3.73) (3.08–4.49)	129 (4.13) (3.33–5.10)	156 (3.68) (3.03–4.48)	93 (2.5) (1.97–3.17)	658 (3.36) (3.07–3.69)	0.905

* Total consumption of benzodiazepines. ^a^ Linear time trend from 2006 to 2016 estimating prevalence of past 30 days.

**Table 3:** Factors associated with nonmedical use of benzodiazepines among native-born and immigrant adolescents in Spain. ESTUDES 2006–2016.

		Native-Born		Immigrants	
		OR (95%CI)	aOR (95%CI)	OR (95%CI)	aOR (95%CI)
Sex	Male	1	1	1	1
	Female	1.62 (1.50–1.76)	1.94 (1.75–2.14)	1.72 (1.41–2.10)	2.37 (1.76–3.19)
Occupational Status of Parents	Both employed	1	1	1	1
	One employed	1.22 (1.11–1.35)	1.13 (1.01–1.28)	1.08 (0.87–1.39)	0.91 (0.67–1.23)
	Both unemployed	1.51 (1.27–1.81)	1.32 (1.07–1.64)	2.85 (2.15–3.79)	2.28 (1.48–3.51)
Alcohol Use in the Past 30 Days	No	1	1	1	1
	Yes	2.14 (1.95–2.34)	1.42 (1.25–1.60)	2.46 (1.99–3.03)	1.75 (1.26–2.43)
Any Cigarette Smoking in the Past 30 Days	No	1	1	1	1
	Yes	2.31 (2.14–2.50)	1.34 (1.19–1.52)	3.13 (2.58–3.81)	1.75 (1.27–2.40)
Marijuana Use in the Past 30 Days	No	1	1	1	
	Yes	2.68 (2.46–2.92)	1.48 (1.30–1.69)	3.75 (3.06–4.59)	1.35 (0.89–2.06)
Any Illicit Psychoactive Drug Use other than Marijuana in the Last 12 Months	No	1	1	1	1
	Yes	8.17 (7.28–9.18)	4.58 (3.92–5.35)	12.46 (9.72–15.97)	6.00 (3.89–9.27)
Perceived Health Risk for Nonmedical Use of Benzodiazepines	Quite a few/many problems	1	1	1	1
	No/few problems	3.57 (3.27–3.90)	2.93 (2.65–3.24)	3.50 (2.75–4.45)	2.67 (1.96–3.61)
Perceived Availability of Benzodiazepines	Impossible/very difficult to obtain	1	1	1	1
	Easy/very easy to obtain	3.87 (3.46–4.33)	3.11 (2.74–3.52)	3.78 (2.83–5.05)	2.77 (2.00–3.83)
Information on Drugs Received at School	Yes	1	1	1	1
	No	1.16 (1.06–1.28)	0.98 (0.87–1.09	1.47 (1.18–1.82)	1.56 (1.15–2.10)

OR = odds ratio; aOR = adjusted OR; CI = confidence interval.

## Data Availability

Not applicable.
